# The *LBP* Gene and Its Association with Resistance to *Aeromonas hydrophila* in Tilapia

**DOI:** 10.3390/ijms151222028

**Published:** 2014-12-01

**Authors:** Gui Hong Fu, Feng Liu, Jun Hong Xia, Gen Hua Yue

**Affiliations:** 1 Molecular Population Genetics & Breeding Group, Temasek Life Sciences Laboratory, 1 Research Link, National University of Singapore, Singapore 117604, Singapore; E-Mails: snow03221@163.com (G.H.F.); liufeng@tll.org.sg (F.L.); xiajunh3@mail.sysu.edu.cn (J.H.X.); 2 Key Laboratory of East China Sea & Oceanic Fishery Resources Exploitation and Utilization, Ministry of Agriculture of China, East China Sea Fisheries Research Institute, Chinese Academy of Fishery Science, Shanghai 200090, China; 3 School of Life Sciences, Sun Yat-Sen University, Guangzhou 266061, China; 4 Department of Biological Sciences, National University of Singapore, 14 Science Drive 4, Singapore 117543, Singapore; 5 School of Biological Sciences, Nanyang Technological University, 60 Nanyang Drive, Singapore 637551, Singapore

**Keywords:** tilapia, gene, SNP, pathogen, association

## Abstract

Resistance to pathogens is important for the sustainability and profitability of food fish production. In immune-related genes, the lipopolysaccharide-binding protein (*LBP*) gene is an important mediator of the inflammatory reaction. We analyzed the cDNA and genomic structure of the *LBP* gene in tilapia. The full-length cDNA (1901 bp) of the gene contained a 1416 bp open reading frame, encoding 471 amino acid residues. Its genomic sequence was 5577 bp, comprising 15 exons and 14 introns. Under normal conditions, the gene was constitutively expressed in all examined tissues. The highest expression was detected in intestine and kidney. We examined the responses of the gene to challenges with two bacterial pathogens *Streptcoccus agalactiae* and *Aeromonas hydrophila*. The gene was significantly upregulated in kidney and spleen post-infection with *S. agalactiae* and *A. hydrophila*, respectively. However, the expression profiles of the gene after the challenge with the two pathogens were different. Furthermore, we identified three SNPs in the gene. There were significant associations (*p* < 0.05) of two of the three SNPs with the resistance to *A. hydrophila*, but not with the resistance to *S. agalactiae* or growth performance. These results suggest that the *LBP* gene is involved in the acute-phase immunologic response to the bacterial infections, and the responses to the two bacterial pathogens are different. The two SNPs associated with the resistance to *A. hydrophila* may be useful in the selection of tilapia resistant to *A. hydrophila*.

## 1. Introduction

Aquaculture is one of the major sources for the supply of animal proteins to humans. However, the success of aquaculture is impeded by the prevalence of infectious diseases [1]. *Streptococcus agalactiae* (*S. agalactiae*) is a beta-hemolytic Gram-positive streptococcus. It is an emerging pathogen that has been associated with considerable morbidity and mortality in fish farms worldwide [2,3]. *S. agalactiae* infections were reported worldwide in a number of fish species, such as, seabream (*Sparus auratus* L.) [4], silver pomfret [2] and tilapia [5]. Besides *S. agalactiae*, *A. hydrophila* is a heterotrophic, Gram-negative, rod-shaped bacterium mainly found in areas with a warm climate. *A. hydrophila* is one of the causative agents of a serious *haemorrhagic septicaemia* that affects a wide range of freshwater fish, such as, zebrafish [6], crucian carp [7] and tilapia [8]. In recent years, the use of antibiotics has partially solved the problem of bacterial infections, but has raised concerns regarding antibiotic residues, environmental pollution and antibiotic resistance development. There is extensive interest in enhancing the resistance to diseases in aquaculture. However, increase of disease resistance through conventional breeding is very difficult. Marker-assisted selection (MAS) may greatly increase the efficiency and effectiveness for breeding compared to conventional breeding [9]. However, before MAS is feasible, markers tightly linked to disease resistance must be identified. Markers associated with disease resistance have been identified in salmon [10] and Japanese flounder [11], leading to substantial increase of disease resistance.

Single nucleotide polymorphism (SNP) markers are abundant and distributed widely and evenly throughout the genome [12]. SNPs serve as suitable markers for linkage mapping, and marker-assisted selection of important traits if they are closely linked to traits [9]. SNPs in the innate immune genes may play a role in determining susceptibility to a range of common diseases, which have an inflammatory component [13]. Previous studies showed that SNPs in some immune-related genes were associated with resistance against bacterial and viral pathogens in some fish species [14,15,16,17]. Thus, analysis of associations between SNPs in candidate genes and resistance against diseases may facilitate the genetic improvement of disease resistance.

Lipopolysaccharide binding protein (LBP) is a soluble acute-phase protein which plays an important role in lipopolysaccharide signaling and innate immunity [18,19]. The protein encoded by the *LBP* gene is involved in the acute-phase immunologic response to gram-negative bacterial infections [20,21]. In fish, LBP also stimulates the non-specific and specific immune response. For example, LBP protected the immune response after immunization with *Aeromonas hydrophila* (*A. hydrophila*) in crucian carp [22], and also influenced the growth and immune stimulation in Atlantic salmon [23]. However, at present, limited information about whether the polymorphisms in the *LBP* gene are associated with disease resistance is available in aquaculture species.

Tilapia is one of the most important good fish species in the world [24]. Its aquaculture faces the challenges of bacterial and viral diseases [24]. We have worked on developing molecular tools to facilitate the breeding of tilapia for growth and disease resistance [17,25,26]. The purposes of this study were to investigate the role of the *LBP* gene in response to bacterial pathogen infections, and to examine whether SNPs in the gene were associated with the resistance to two major bacterial pathogens of tilapia: *S. agalactiae* and *A. hydrophila*. We identified the cDNA and genomic DNA sequence of the *LBP* gene. We determined its expression profiles after challenge with the two pathogens at different time-points. We identified significant associations between the SNPs in the *LBP* gene and the resistance to *A. hydrophila*, but not to *S. agalactiae* in tilapia. The SNPs associated with the resistance to *A. hydrophila* may be useful in the selection of tilapia resistant to *A. hydrophila*.

## 2. Results

### 2.1. Sequence of cDNA and Genomic DNA of the Lipopolysaccharide Binding Protein (LBP) Gene

We identified the cDNA and genomic structure of the *LBP* gene using bioinformatics tools and confirmed the sequences of the *LBP* gene by PCR and sequencing PCR products. The full-length cDNA (1901 bp) of the *LBP* gene contained a 1416 bp open reading frame, encoding 471 amino acid residues. We also analyzed the genomic sequence of the LBP gene. It was 5577 bp, comprising 15 exons (120, 110, 111, 132, 156, 64, 61, 93, 176, 60, 169, 67, 43, 61 and 78 bp, respectively) and fourteen introns (123, 124, 194, 757, 85, 127, 119, 183, 101, 1128, 224, 85, 86, 227 and 112 bp, respectively).

### 2.2. Expression Profiles of the LBP Gene in Normal Individuals

**Figure 1:**
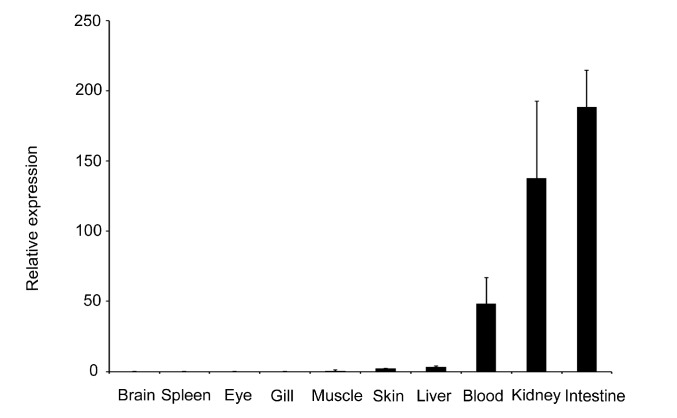
Expressions of the lipopolysaccharide binding protein (*LBP*) gene in different tissues of normal tilapia. The relative expression fold- was calculated with the ΔΔ*C*_t_ method using β-actin as a reference gene. The expression in blood, kidney and intestine was significantly higher (*p* < 0.01) than in other tissues.

To determine the transcriptional level of the *LBP* gene in various tissues of normal fish at the age of three months, qRT-PCR was performed. The expression levels of the gene in different tissues were compared with the level of expression in the brain (Figure 1). The *LBP* gene was constitutively expressed in all examined tissues. The highest mRNA expression was detected in the intestine, kidney and blood. The expression levels in liver and skin were moderate. The lowest mRNA expression was detected in the brain.

### 2.3. Expression Profiles of the LBP Gene in Individuals Infected with Bacteria

**Figure 2:**
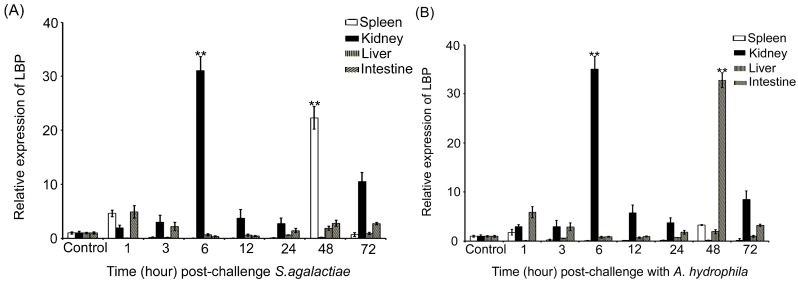
Expression levels of the *LBP* gene in four tissues of tilapia after a challenge with bacterial pathogens *S. agalactiae*, and *A. hydrophila*, respectively. The relative expression after the challenge with bacterial pathogens *S. agalactiae* (**A**) and *A. hydrophila* (**B**), respectively was calculated with the ΔΔ*C*_t_ method using β-actin as a reference gene. The relative expression fold at each time point was compared to that of PBS-injected samples as control. ** indicates that the expression was significantly (*p* < 0.01) higher than in other tissues.

In order to understand the expression profiles of the *LBP* gene after challenges with *S. agalactiae* and *A. hydrophila*, qRT-PCR analysis was used to examine the relative expressions of the *LBP* gene in the liver, spleen, kidney and intestine (Figure 2). In the group challenged with *S. agalactiae*, the *LBP* transcript increased up to 22.3-fold at 48 h post-challenge, and decreased at other time-points compared to that of the control in the spleen. In the kidney, the *LBP* transcript increased 31.1-fold at 6 h post-challenge compared to the control group, and then was decreased from 12 to 48 h. In the liver, the highest expression level increased 1.9-fold at 48 h post-challenge compared the control group. In the intestine, the *LBP* transcript increased 5.0-fold at 1 h post challenge, and decreased at other time-points. The changes in expression of *LBP* mRNA were also investigated after the challenge with *A. hydrophila*. The *LBP* transcript level was induced significantly with a 32.8-fold increase in the intestine 48 h post the challenge compared to the control group. The transcript level was induced significantly with a 35.1-fold increase in the kidney 6 h post the challenge in the kidney compared to the control group. However, in the liver and spleen, the transcription level was weakly induced compared to the intestine and kidney.

### 2.4. Identification of Single Nucleotide Polymorphisms (SNPs) in the LBP Gene

**Figure 3:**
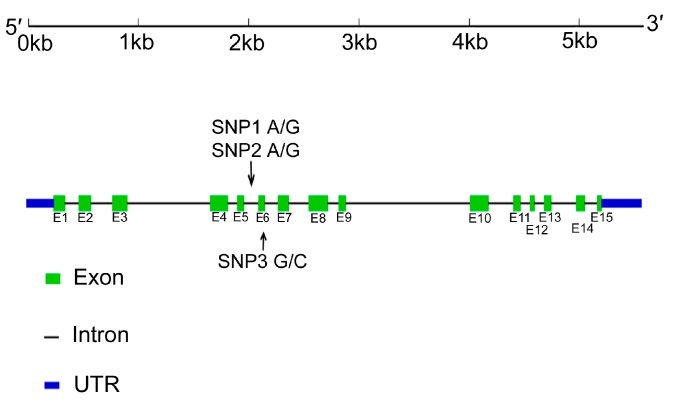
Genomic structure of the *LBP* gene and positions of known single nucleotide polymorphisms (SNPs) in tilapia.

We identified three SNPs in the *LBP* gene in 10 unrelated individuals by sequencing a part of the gene. One SNP (*i.e.*, SNP3) was located in exon 6 and two SNPs (*i.e.*, SNP1 and SNP2) were located in intron 5 of genomic DNA. The SNP1 g.1836 A>G and SNP2 g.1894 A>G were located in intron 5. The SNP3 g.1948 G>C was located in exon 6, which resulted in an amino acid change (Figure 3).

### 2.5. Identification of Associations between SNPs and Disease Resistance/Susceptibility to Bacteria in Tilapia

We found no significant association (*p* > 0.05) between all SNPs and the resistance to *S. agalactiae* disease. There were no significant differences (*p* > 0.05) in allele frequencies of the three SNPs in the surviving and dead fish (Table 1).

Significant associations (*p* < 0.005) between genotype distributions in SNP1 and SNP 3 of the *LBP* gene and the resistance to the *A. hydrophila* were found (Table 1). However, there is no significant association (*p* > 0.05) of genotypes at SNP2 with the resistance to *A. hydrophila* (Table 1). We also analyzed the allele frequencies between the susceptible and resistant individuals. The results showed that SNP1 showed a significant association (*p* < 0.005) with the resistance to the *A. hydrophila*. In contrast, the allele frequencies at SNP2 and SNP3 were not significantly associated (*p* > 0.05) with the resistance to the *A. hydrophila*.

**Table 1:** SNPs in the *LBP* gene in susceptible and resistant groups of tilapia.

SNP	Number (%)			Allele Number (%)			
Genotypes	Susceptible	Resistant	*X*^2^	Allele	Susceptible	Resistant	*X*^2^
*S. agalactiae*							
SNP1							
AA ( *n* = 332)	200 (66)	132 (73)	2.03	A	202 (66)	114 (70)	1.12
AG ( *n* = 150)	102 (34)	48 (27)	( *p* > 0.05)	G	102 (34)	48 (30)	( *p >* 0.05)
GG ( *n* = 0)	0 (0)	0 (0)					
SNP2							
AA ( *n* = 8)	6 (2)	2 (1)	1.14	A	86 (20)	38 (16)	2.26
AG ( *n* = 58)	40 (13)	18 (10)	( *p >* 0.05)	G	340 (80)	196 (84)	( *p >* 0.05)
GG ( *n* = 420)	260 (85)	160 (89)					
CC ( *n* = 18)	14 (5)	4 (2)	1.19	C	134 (28)	76 (26)	0.29
CG ( *n* = 96)	60 (20)	36 (20)	( *p* > 0.05)	G	348 (72)	212 (74)	( *p* > 0.05)
GG ( *n* = 368)	228 (75)	140 (78)					
*A. hydrophila*							
SNP1							
AA ( *n* = 354)	188 (71)	166 (88)	14.61	A	340 (69)	206 (82)	20.70
AG ( *n* = 96)	76 (59)	20 (11)	( *p <* 0.005)	G	152 (31)	44 (18)	( *p* < 0.05)
GG ( *n* = 2)	0 (0)	2 (1)					
SNP2							
AA ( *n* = 8)	8 (3)	0 (0)	2.97	A	52 (16)	32 (13)	0.96
AG ( *n* = 38)	22 (8)	16 (8)	( *p* > 0.05)	G	278 (84)	206 (87)	( *p* > 0.05)
GG ( *n* = 408)	234 (89)	174 (92)					
SNP3							
CC ( *n* = 28)	8 (3)	20 (10)	18.34	C	100 (25)	72 (27)	0.50
CG ( *n* = 72)	46 (18)	26 (14)	( *p* < 0.005)	G	300 (75)	196 (73)	( *p* > 0.05)
GG ( *n* = 352)	208 (79)	144 (76)					

*p* < 0.005 is considered to be statistically significant.

### 2.6. Associations between SNPs in the LBP Gene and Growth Traits

The three SNPs were found to be polymorphic in a family with 290 individuals where the growth traits of individuals were measured. The analysis of associations between the three SNPs and growth traits revealed that all three SNPs were not significantly (*p* > 0.05) associated with growth traits (Supplementary Table S1).

### 2.7. In-Silico Mapping of the LPB Gene to Linkage Group 20

Through *in-silico* mapping, the *LBP* gene was mapped to linkage group 20 of the Nile tilapia genome. Around the location of the *LBP* gene, the transporter genes, including vesicular inhibitory amino acid transporter, actin-related protein 5, protein slowmo homolog 2 and ATP synthase subunit epsilon, were also located.

## 3. Discussion

LBP is an acute-phase protein that initiates an immune response after recognition of bacterial LPS [18,19]. We cloned the full-length cDNA of the *LBP* gene in tilapia. The gene encoded 471 amino acids, which is similar to the *LBP* genes in other vertebrates [18,27,28,29]. The deduced amino acid sequence of the *LBP* gene was highly homologous with that reported for fish, rat and human *LBP* [27,30,31]. The *LBP* gene consisted of 15 exons separated by two relatively large introns (0.8–1.1 kb) and twelve small introns (0.13–0.23 kb). Comparison of the genomic DNA structure of *LBP* from tilapia with those from other animals [27,32,33] indicates that the *LBP* gene has largely maintained its genomic structure during evolution.

We investigated the expression profiles of the *LBP* gene in normal fish at the age of three months using qRT-PCR. The *LBP* gene was expressed in all 10 tissues examined, and the highest expression was in the intestine and kidney. Similar expression patterns were reported in common carp [34], sweetfish *Plecoglossus altivelis* [29] and rock bream *Oplegnathus fasciatus* [28]. These results suggest that the *LBP* gene plays a wide range of roles in vertebrates. The kidney is an important immune organ in fish [35], and recent research showed that the intestine is also an important immune organ [36,37]. Therefore, the high expression levels of the *LBP* gene in these two organs suggest that the *LBP* gene plays an important role in the immune system in tilapia, which is in agreement with the findings in mammals [19,32].

We further analyzed the expression profiles of the *LBP* gene in immune-related organs in fish challenged with *S. agalactiae* and *A. hydrophila*, which are the major pathogens that resulted in significant economic losses in aquaculture [38,39]. The expression levels of the *LBP* gene were up-regulated in kidney, spleen, liver and intestine after challenge with *S. agalactiae* and *A. hydrophila*. The expression levels of the *LBP* gene in *S. agalactiae*-infected fish were significantly increased in the kidney (up to 31.0-folds) and spleen (up to 22.0-folds) compared to the intestine (5.0-folds) and liver (1.9-folds). The expression levels of the *LBP* gene in *A. hydrophila*-infected fish were increased in the kidney (35.1-folds) and intestine (32.8-folds) compared to the spleen and liver. Our results suggest that the *LPB* gene is involved in the acute-phase immunologic response to bacterial infections. The different patterns of the expression profiles of the *LBP* gene in fish challenged with two different bacterial pathogens indicate different responses of the gene to the two bacterial pathogens. *A. hydrophila* is a Gram-negative pathogen, which possesses a membrane component known as LPS. LBP is a specific protein that binds with LPS and is responsible for eliciting an immune response against LPS in vertebrates [34,40]. Therefore, the response of the *LBP* genes to the Gram-negative *A. hydrophila* is easily understandable. Certainly, it is also possible that the localization of these two bacteria in different tissues during infection could also be a reason for the different expression levels of the *LBP* gene in different tissues infected with the two bacteria. However, in our study, we found that the *LBP* gene also responded to the challenge of the Gram-positive bacterium *S. agalactiae*. *S. agalactiae* does not possess the LPS in its membrane. Therefore, the pathways involving the *LBP* gene in response to Gram-positive and negative bacteria could be different. A previous study showed that *LBP* also mediated cytokine induction caused by compounds derived from Gram-positive bacteria, including lipoteichoic acid and peptidoglycan fragments [41]. Further study on pathways involving the *LBP* genes in fish may bring new insights on the functions of the *LBP* gene in the resistance to bacterial pathogens.

We also examined whether the polymorphisms in the *LBP* gene were associated with the resistance to the two bacterial pathogens *A. hydrophila* and *S. agalactiae* using association analysis. Three SNPs were detected in part of the *LBP* gene. We detected significant associations in genotype distributions or allele frequencies of the SNP1 and SNP3 in the *LBP* gene and the resistance to *A. hydrophila*. This result suggests that the *LBP* gene itself plays a role in the resistance to *A. hydrophila*, or the *LBP* gene is linked to a gene or several genes, which play a role in the resistance to *A. hydrophila*. However, with our current data, it is impossible to dissect these two possibilities. Using QTL mapping in segregating families, it is possible to know whether the associations were due to the *LBP* gene itself or its linked genes. The *LBP* gene was located in LG20, and DNA sequences flanking the *LPB* gene are available in public domains (e.g., http://www.ensembl.org/Oreochromis_niloticus/Info/Index). Around the linkage location of the *LBP* gene, there are transporter genes, including vesicular inhibitory amino acid transporter, actin-related protein 5, protein slowmo homolog 2 and ATP synthase subunit epsilon. It is easy to get DNA markers flanking the *LBP* gene for QTL mapping. We noted that there was no association between SNPs in the *LBP* gene and the resistance to *S. agalactiae*. This may be because *S. agalactiae* is a beta-hemolytic and heterotrophic Gram-positive streptococcus, while *A. hydrophila* is a heterotrophic, Gram-negative bacteria. Certainly, it is also possible that there are more than the three SNPs in the *LBP* gene, and the use of three SNPs may not be able detect the association. Analysis of all SNPs in the *LBP* gene may give more information about whether the *LBP* gene is associated with the resistance to the Gram-positive bacterial pathogen *S. agalactiae.*

In conclusion, we identified and characterized the *LBP* gene in tilapia for the first time. The *LBP* gene was expressed in all tissues examined, but was highly expressed in the intestine and kidney. The different patterns of the expression profiles of the *LBP* gene in the kidney, spleen, liver and intestine of fish challenged with two different bacterial pathogens (*i.e.*, *S. agalactiae* and *A. hydrophila*) indicate different responses of the gene to the two pathogens. We found significant associations between two SNPs in the *LBP* gene and the resistance against *A. hydrophila*, but not against *S. agalactiae*. The two SNP markers associated with resistance against *A. hydrophila* may be used in the selection of fishes resistant to *A. hydrophila*.

## 4. Experimental Section

### 4.1. Fish and Ethics Statement

Tilapia individuals were cultured in a fish farm in Singapore. One hundred and fifty-two individuals at the age of 60-days post-hatch (dph) with an average body weight of 25 ± 2.68 g were transported to a large tank containing 500 L seawater located in the animal house of our institute three weeks before the experiment. The fish were maintained in the large tank, and were fed twice daily with pellet feed (Biomar, Nersac, France). All handling of fish was conducted in accordance with the guidelines on the care and use of animals for scientific purposes set up by the Institutional Animal Care and Use Committee (IACUC) of Temasek Life Sciences Laboratory (TLL), Singapore. The IACUC of TLL has specially approved this study within the project “Breeding of tilapia resistant to microbe pathogens” in April 2012 (approval number: TLL (F)-12-004).

### 4.2. Identification of cDNA and Genomic DNA Sequences of the LBP Gene

The cDNA sequence of the *LBP* gene in tilapia was downloaded from NCBI database (DNA sequence Genbank No. XR_270272) and genomic DNA sequence was derived by BLAST the cDNA sequence against the assembled whole genome sequence of a Nile tilapia individual. The cDNA and genomic DNA sequences were used to design primers (Table 2) to confirm the cDNA and genomic DNA sequences, identify SNPs and analyze their expression levels in different tissues, respectively.

**Table 2:** Primers used for amplifying genomic DNA of the *LBP* gene, SNP identification and expression analysis in Mozambique tilapia.

Name	Primer Sequence (5'–3')	T (°C)	Product Length (bp)	Application
*LBP-RT-F1*	GGCGCAGCTGGGGAAAGAA	60	269	qRT-PCR
*LBP-RT-R1*	TGGGGACATCAGTGAGAGGAAGG			
*β-actin F1*	TGACCCAGATCATGTTCGAGAC	60	253	qRT-PCR
*β-actin R1*	GTGGTGGTGAAGGAGTAGCC			
*LBP-G-F1*	ACTTCTCAGTGACACAGGAAATTA	57	1827	Genomic DNA
*LBP-G-R1*	CTGGCTCCACCATGAAATTCTAT			
*LBP-G-F3*	GAAAAGCAAAACCAACCAGCTTG	57	2004	Genomic DNA
*LBP-G-R3*	TATGACAGAAGTTGTTTTTAATCCT			
*LBP-G-F2*	GGCGCAGCTGGGGAAAGAAGCTGA	57	1902	SNP detection
*LBP-G-R2*	TAGTTATAGGCATAGTATATGTTTG			

### 4.3. RNA Extraction and Quantitative Real-Time RT-PCR

Total RNA was isolated from five individuals at the age of two months using Trizol reagent (Invitrogen, Carlsbad, CA, USA) according to the manufacturer’s instructions. Ten tissues were collected to examine expression profiles of the *LBP* gene. These 10 tissues were the spleen, blood, brain, gill, intestine, liver, skin, kidney, muscle and eye. First strand cDNA was synthesized using Fermentas cDNA Synthesis Kit (Fermentas, Pittsburg, PA, USA) from total RNA (1.0 μg). We used quantitative real-time PCR (qRT-PCR) with the primer pair LBP-RT-F1R1 (Table 2) to analyze the mRNA distribution in these samples. We used β-actin as the reference gene (Primers β-actin F1 and R1, Table 2). PCR amplification was performed in a total volume of 20 μL containing 1× MaximaTM SYBR Green qPCR Master Mix (Fermentas, Pittsburg, PA, USA), 0.2 μL (10 μM) of each primer and 1 μL cDNA generated from RNA template. The cycling conditions consisted of an initial single cycle of 10 min at 95 °C followed by 40 cycles of 15 s at 95 °C, 30 s at 57 °C and 20 s at 72 °C. PCRs were performed in triplicates. The transcription level of the *LBP* gene was analyzed using the ΔΔ*C*_t_ method [42].

### 4.4. Bacterial Challenge and Sampling Tissues

*Streptococcus agalactiae* strain ATCC^®^ 624™. The bacterial culture was grown for 24 h in Brain-heart infusion broth (BHI, Oxoid, UK) at 37 °C with continual shaking. The concentration of incubated bacteria was 1.0 × 10^8^ CFU/mL. *Aeromonas hydrophila* strain used was ATCC^®^ 7966™. The bacterial culture was grown for 24 h in Trypticase soy broth (BHI, Oxoid, UK) at 30 °C with continual shaking. The concentration of incubated bacteria determined by standard direct plate count was 1.0 × 10^8^ CFU/mL. The bacteria for experiment were washed with PBS before injection to tilapia.

One hundred and five disease-free fish weighing 25.0 ± 0.20 g were used in the bacterial infection experiment. Fish were divided into three groups (35 individuals in each group). The conditions were the same among tanks, and the fish were randomly distributed into different tanks. Three groups were cultured in three big tanks. Since our pretest showed that LD_50_ values of tilapia infected with *S. agalactiae* and *A. hydrophila* were 1.0 × 10^6^ CFU/mL, the first group received individual intraperitoneal injection with 0.1 mL of *S. agalactiae* of 1.0 × 10^6^ CFU/mL. The second group received intraperitoneal injection with 0.1 mL of *A. hydrophila* of 1.0 × 10^6^ CFU /mL. The third group injected just with PBS served as control. Sampling was performed 1, 3, 6, 12, 24, 48 and 72 h after challenge, with five fish in each group. Liver, spleen, kidney and intestine were sampled and kept in liquid nitrogen for total RNA extraction.

To analyze whether SNPs in the *LBP* gene were associated with the resistance to the infections of the two bacterial pathogens, nine hundred and forty two disease-free tilapia individuals (weighing 25.8 ± 0.8 g) were used in this experiment. Fish were divided into two groups. Since the challenge with bacterial pathogens by immersion reflects natural infection, one group of tilapia was challenged with *S. agalactiae* while the other group was challenged with *A. hydrophila* by immersion exposure with approximately 10^5^ viable bacteria mL^−1^ tank water for 2 h. Dissolved oxygen levels (>5.7 mg/L) were maintained throughout the exposure procedure. After an exposure period of 2 h, the fish were removed and placed into their respective tanks and maintained at 28 °C in fresh water. All the fish were observed daily to survey the mortality, and samples were collected until the termination of the experiment at 14-days after challenge. Tilapia that died in the first 72 h post-challenge were classified into the susceptible group for their high sensitivity to bacteria, while the fish that survived over 14-days post-challenge were considered as a resistant group. Fin clips of each fish were collected for DNA extraction. DNA was isolated using a method developed by us [43].

### 4.5. Identification and Genotyping of SNPs in the LBP Gene

To identify SNPs in the *LBP* gene, one pair of primers (*LBP-G-F2* and *R2*, Table 2) were designed to amplify a part of the genomic DNA of the tilapia *LPB* gene using PrimerSelect (DNAstar, Madison, WI, USA). PCR was conducted as described above. The genotyping of three identified SNPs were conducted by directly sequencing PCR products using an ABI 3730xl sequencer (ABI, Foster City, CA, USA). SNP genotypes were analyzed using the software Sequencher (Genecodes, Ann, Arbor, MA, USA). The genotypes were used to examine whether they were associated with disease resistance.

### 4.6. Association Analysis of SNPs with Resistance to the Infection of Two Bacterial Pathogens

In order to examine whether the SNPs in the *LBP* gene were associated with resistance to *S. agalactiae* and *A. hydrophila* disease, we genotyped the surviving and dead individuals after challenge with two bacterial strains, respectively. We collected fin clips from 306 and 266 individuals susceptible to *S. agalactiae* and *A. hydrophila* respectively. One hundred and eighty and 190 individuals resistant to the *S. agalactiae* and *A. hydrophila* respectively were used as controls. SNPs in the *LBP* gene were genotyped in all 942 fish. The Statistical Program for Social Science (SPSS) (SPSS Inc., Chicago, IL, USA) version 1.0 was used for data analysis. The frequencies of genotypes and alleles were compared in the susceptible and resistant individuals collected from the infections by two bacterial pathogens using Chi-square test.

### 4.7. Association Analysis of SNPs in the LBP Gene with Growth Traits

To analyze associations between growth traits and SNPs in the *LBP* gene, a population, including 270 tilapia individuals was used. All fish were raised in the fish facility of Temasek Life Sciences laboratory. Individuals were raised communally in big tanks and maintained on strict feeding regimes until 140 dph. Growth traits, including body weight, standard and total length data were measured at 140 dph. Fulton’s condition factor K (KTL: condition factor K based on total length, and KSL: condition factor K based on standard length) was calculated based on body weight (BW), Total length (TL) and Standard length (SL). Fin clips were sampled from each individual, and stored in 100% ethanol for subsequent DNA extraction with the method described previously [43]. All individuals were genotyped by PCR amplification and sequencing of the genomic DNA of the *MCP*-8 gene as described above. Allele frequency for all the SNPs was statistically assessed using the Haploview software package [44]. Association between MCP-8 SNPs and quantitative traits were analyzed with SPSS 19.0 program.

### 4.8. Mapping the LBP Gene to the Genome of Tilapia

To map the *LBP* gene to the genome of tilapia, we blasted the ORF of the *LBP* gene against the assembled Nile tilapia genome sequence in the public domain (http://www.ensembl.org/Oreochromis_niloticus/blastview). The hit with the lowest E value was regarded as the position of the gene in the tilapia genome. The scaffold containing the *LBP* gene was derived and scanned for genes located near the *LBP* gene using web-based software GENSCAN.

## Abbreviations

LBP: lipopolysaccharide-binding protein
*S. Agalactiae*: *Streptcoccus agalactiae*
*A. Hydrophila*: *Aeromonas hydrophila*
SNP: Single nucleotide polymorphism
qRT-PCR: Quantitative real-time PCR

## Supplementary Material

Supplementary table can be found at http://www.mdpi.com/1422-0067/15/12/22028/s1.
